# Coordinating across correctional, community, and VA systems: applying the Collaborative Chronic Care Model to post-incarceration healthcare and reentry support for veterans with mental health and substance use disorders

**DOI:** 10.1186/s40352-019-0099-4

**Published:** 2019-12-12

**Authors:** Bo Kim, Rendelle E. Bolton, Justeen Hyde, B. Graeme Fincke, Mari-Lynn Drainoni, Beth Ann Petrakis, Molly M. Simmons, D. Keith McInnes

**Affiliations:** 1 VA Center for Healthcare Organization and Implementation Research, Bedford/Boston, MA USA; 2 Harvard Medical School, Boston, MA USA; 3 Brandeis University The Heller School for Social Policy and Management, Waltham, MA USA; 4 Boston University School of Medicine, Boston, MA USA; 5 Boston University School of Public Health, Boston, MA USA; 6 RAND Corporation, Boston, MA USA

**Keywords:** Continuity of care, Mental health, Qualitative research, Substance abuse, Veterans

## Abstract

**Background:**

Between 12,000 and 16,000 veterans leave incarceration annually. As is known to be the case for justice-involved populations in general, mental health disorders (MHDs) and substance use disorders (SUDs) are highly prevalent among incarcerated veterans, and individuals with MHDs and SUDs reentering the community are at increased risk of deteriorating health and recidivism. We sought to identify opportunities to better coordinate care/services across correctional, community, and VA systems for reentry veterans with MHDs and SUDs.

**Methods:**

We interviewed 16 veterans post-incarceration and 22 stakeholders from reentry-involved federal/state/community organizations. We performed a grounded thematic analysis, and recognizing consistencies between the emergent themes and the evidence-based Collaborative Chronic Care Model (CCM), we mapped findings to the CCM’s elements – work role redesign (WRR), patient self-management support (PSS), provider decision support (PDS), clinical information systems (CIS), linkages to community resources (LCR), and organizational/leadership support (OLS).

**Results:**

Emergent themes included (i) WRR – coordination challenges among organizations involved in veterans’ reentry; (ii) PSS – veterans’ fear of reentering society; (iii) PDS – uneven knowledge by reentry support providers regarding available services when deciding which services to connect a reentry veteran to and whether he/she is ready and/or willing to receive services; (iv) CIS – lapses in MHD/SUD medications between release and a first scheduled health care appointment, as well as challenges in transfer of medical records; (v) LCR – inconsistent awareness of existing services and resources available across a disparate reentry system; and (vi) OLS – reentry plans designed to address only immediate transitional needs upon release, which do not always prioritize MHD/SUD needs.

**Conclusions:**

Applying the CCM to coordinating cross-system health care and reentry support may contribute to reductions in mental health crises and overdoses in the precarious first weeks of the reentry period.

## Background

Approximately 181,500 veterans are estimated to be incarcerated in US state and federal correctional facilities (Bronson et al. 2015). This may likely be an underestimate, given potential stigma in reporting and inconsistent methods utilized by criminal justice agencies in keeping track of this number (Baldwin 2016). Justice-involved veterans comprise nearly a tenth of all arrestee, jail, prison, and community-supervision populations (Blue-Howells et al. 2013). Annually, 12,000 to 16,000 veterans are released from incarceration (Homeless Services Cube 2014). Their reentry into the community requires coordination across multiple health care and support services (Visher and Travis 2011). The Department of Veterans Affairs (VA)‘s Health Care for Reentry Veterans (HCRV) program links incarcerated veterans to VA and community health care services (VA HCRV Program Handbook 2014). However, given the program’s primary focus on short-term post-incarceration case management (US Department of Veterans Affairs 2019b), many veterans may not receive sufficient longer-term support, including assistance attending appointments for physical/behavioral health care, housing, and employment (Wortzel et al. 2012). Homelessness and criminal justice recidivism may result when such support is lacking (Baillargeon et al. 2009; Meyer et al. 2011; Swan 2015). The period of leaving incarceration is a particularly vulnerable time for veterans with mental health disorders (MHDs) and substance use disorders (SUDs), as they are likely to experience disruption in established mental health and SUD treatment/medications (Baillargeon et al. 2009; Meyer et al. 2011; Massoglia and Schnittker 2009; Hartwell et al. 2013). Rarely do they leave incarceration with comprehensive plans for coordinating treatment and additional supports required for successful reentry (Draine and Herman 2007).

Reentry efforts have been, and continue to be, a major focus for correction practitioners and researchers, especially given that 93% of incarcerated individuals in the US are expected to be released during their lifetime (Berghuis 2018). Mallik-Kane and Visher’s seminal 2008 study of the reentry process, through a representative sample of 1100 individuals, noted (i) prevalent chronic and comorbid health conditions, (ii) treatment discontinuity, (iii) challenges with housing and employment, and (iv) recidivism as some of the key issues facing individuals leaving incarceration. Both prior to and following Mallik-Kane and Visher’s 2008 study, reentry efforts have been actively examined, which have further highlighted these issues and noted the limited engagement that individuals leaving incarceration have with health care and social services (Kendall et al. 2018). Effectiveness of reentry support programs, both need-specific [e.g., housing (Miller and Ngugi 2009), employment (Newton et al. 2018)] and multimodal (Duwe 2012), have been studied, and the number of systematic reviews are few but on the rise (Berghuis 2018). A recent 2018 review by Moore and colleagues points to the need for additional evidence regarding which interventions are effective, and key findings from both Kendall and colleagues’ and Berghuis’ 2018 reviews emphasize the importance of coordinating care from pre-release to post-release for individuals leaving incarceration.

As is discussed within Finlay and colleagues’ recent review of health and healthcare of military veterans involved in the criminal justice system (Finlay et al. 2019), although veterans notably comprise approximately 8% of US’ incarcerated population (Bronson et al. 2015), they have not been a focus of research studies until recently, unlike other vulnerable populations (e.g., women, older adults). Justice-involved veterans may have different healthcare needs than other justice-involved populations (Backhaus et al. 2016) – e.g., higher rate of mental health concerns and more prevalent intravenous drug use (Blodgett et al. 2015). Meeting the needs of justice-involved veterans can therefore contribute to safer communities (Finlay et al. 2019). At the same time, veterans also face many similar challenges upon reentry as other justice-involved populations, such as securing housing, finding employment, and balancing healthcare with other competing needs (McDonough et al. 2015; Mallik-Kane and Visher 2008). Hence, the importance of studying the veteran population is two-fold. First, as veterans are treated in increasingly diverse settings with legislative changes in recent years such as the VA MISSION Act (Reddy et al. 2019), findings can inform the various settings to be ready to meet veteran-specific needs. Second, given the many overlapping needs of veterans and other vulnerable populations, lessons learned regarding how the VA can best meet veterans’ needs are highly applicable to many healthcare systems beyond the VA, especially ones that are becoming integrated with the development of accountable care organizations (Fullerton et al. 2016; Wu et al. 2016).

For coordination within the clinical care realm, both VA and non-VA clinics have increasingly adopted the evidence-based Collaborative Chronic Care Model (CCM), structuring chronic mental/physical care to be anticipatory, continuous, and patient-centered (Von Korff et al. 1997; Wagner et al. 1996; Miller et al. 2013; Woltmann et al. 2012) – e.g., the CCM underlies the patient-centered medical home (Wagner et al. 2012). Reentry individuals often have needs beyond clinical care (e.g., housing, vocational training, and legal services), which require coordination across correctional, community, and health care systems. Fundamentally, little research has examined veterans’ health/psychosocial needs through the lens of an organizational and service delivery model that seeks coordination/integration of services. There is pressing need for such work due to this population’s high MHD- and SUD-related morbidity (Finlay et al. 2017), with about 50% of veterans incarcerated in state prisons reporting experienced symptoms of MHDs, and about 75% reporting drug use prior to incarceration (Noonan and Mumola 2007).

Therefore, our objective was to examine the challenges of reentry veterans with MHDs and SUDs, based on qualitative interviews with both (i) reentry veterans and (ii) providers of health care and support services to these veterans (referred to as “stakeholders” here onwards). Our data analysis (using codes inductively developed from the data without reference to the CCM; described in further detail in the Methods section) identified challenges that are consistent with, and thus potentially addressable through, the six core elements of the CCM – (i) work role redesign, (ii) patient self-management support, (iii) provider decision support, (iv) clinical information systems, (v) linkages to community resources, and (vi) organizational/leadership support (Bodenheimer et al. 2002a, b; Coleman et al. 2009; Tsai et al. 2005). Definitions for each of the six CCM elements are provided in Table 1.

**Table 1:** Core elements of the Collaborative Chronic Care Model (CCM) (Bodenheimer et al. 2002a, b; Coleman et al. 2009; Tsai et al. 2005; Bauer et al. 2016)

Core element	Definition
Work role redesign	Structuring care tasks of multiple clinical staff in relation to one another, such that patient needs are collaboratively anticipated and met in a timely manner
Patient self-management support	Strengthening patient’s ability to effectively contribute to his/her own wellbeing even during times when he/she is not in direct contact with care providers
Provider decision support	Furnishing relevant information to care providers about available services, treatments, and expertise, to help them best address patient’s care needs
Clinical information systems	Activating feedback systems to share data and monitor both how care is being delivered and how patient is responding
Linkages to community resources	Connecting patient to care resources beyond those available from his/her main clinical setting
Organizational / Leadership support	Championing of clinic’s change efforts toward more CCM-oriented care (i.e., care exhibiting core CCM elements) by clinic’s organizational leaders

The CCM has advantages as a framework for suggesting improvements to reentry services, in that it is already a familiar model to many clinical and management staff and leaders in health care systems. Many health care providers already carry out CCM-aligned processes to coordinate care. Their practical hands-on experience with the CCM may enhance the model’s extension into the realm of reentry support. Further, for many clinicians, understanding an individual’s life circumstances beyond the immediate diagnosis has become common practice, where they are increasingly aware of how these circumstances affect patient care and patient self-management. Applying this perspective of the whole person embedded in a community (beyond an individual’s identity as a patient) to reentry individuals is likely a natural extension for clinicians, and will help clinicians contribute to effectively bridging clinical care and reentry support.

In this paper, we describe our qualitative analytical approach, then share our findings regarding challenges in coordinating health care and reentry support for veterans with MHDs and SUDs leaving incarceration. We discuss implications of our findings for how the CCM and its extensive evidence base can serve as a useful guide for coordinating medical and other services for individuals during reentry, pointing out the parallels between the challenges of reentry and health care delivery.

## Methods

The study was submitted to the Institutional Review Board (IRB) at the Edith Nourse Rogers Memorial Veterans Hospital (Bedford, Massachusetts, USA), which determined it was a quality improvement project as per VA handbook 1200.05. The need for continued IRB review was waived.

### Study context

Data for this paper were drawn from qualitative interviews that were conducted for a multi-year pilot initiative – the Post-Incarceration Engagement (PIE) project, which is implementing peer-support for reentry veterans (Simmons et al. 2017). The PIE project aims to (i) conduct contextual analysis to identify VA and community reentry resources, and describe how reentry veterans use them, (ii) implement peer-support, in one state, to link reentry veterans to VA primary care, mental health, and substance use disorder services, then (iii) port the peer-support intervention to another, geographically and contextually different state (Simmons et al. 2017). This specific study fell under the first aim of the larger PIE project (i.e., the project’s formative stage), for which contextual analysis was performed through in-depth interviews to understand the experience of reentry from multiple perspectives, including planning for, access to, and utilization of health care and other services by veterans following their release from incarceration.

### Participant recruitment

Stakeholder participants from Massachusetts organizations involved in the reentry process were recruited using a combination of snowball and purposive sampling strategies (Wood and Christy 1999; Chang et al. 2009), in order to help ensure that we gather data on each of federal, state, and community organization perspectives. We focused on Massachusetts to have the interviews directly inform the aforementioned PIE project’s efforts to implement peer-based services in Massachusetts to support veterans’ reentry after leaving incarceration (Simmons et al. 2017). We began by identifying potential participants at relevant reentry-involved organizations through conversations with VA’s homelessness programs and HCRV staff (Blue-Howells et al. 2013) familiar with available reentry support programs. Additional organizations were identified by interview participants, and, with the assistance of the leadership of each new organization, we identified staff to be interviewed who could increase the breadth of representation of reentry support programs. For recruiting veteran participants, we used a snowball sampling strategy by asking our stakeholder participants to help identify veterans who had been released from incarceration less than six months prior to the interview.

### Data collection

Between March and September 2016, trained qualitative interviewers conducted interviews, in-person or by phone, with 16 veterans and 22 stakeholders involved in the reentry process, representing federal, state, and community organization perspectives. Interviews were audio-recorded and transcribed verbatim. Recording was not possible with some correctional facility staff, in which case detailed notes were taken by the interviewer(s). Specifically, six interviewers conducted interviews (one or two per interview). Three of the 38 interviews were conducted on the phone instead of in person, and nine were not recorded. Each interview lasted approximately 60 min, and the interviews were semi-structured. The topics addressed by the interviews included experiences and perceptions of (i) needs of veterans leaving incarceration, (ii) process of reentry planning and follow-up coordination efforts, (iii) existing and desirable health care and support services, and (iv) challenges and gaps in coordination across the correctional, community, and VA systems involved in veterans’ reentry after leaving incarceration.

### Data analysis

(a) Overview: We performed a grounded thematic analysis, with codes inductively developed from the data (Miles and Huberman 1994) without reference to the CCM. We aligned closely to Guest et al. (2012)'s four steps in undertaking thematic analysis, as outlined by Chapman et al. (2015) for applications to healthcare research – (i) getting acquainted with data, (ii) recognizing emergent themes, (iii) subdividing/combining and grouping themes into categories, and (iv) conceptualizing the model that interrelates the themes. We examined themes emerging from data segments coded with the “mental health” code and/or the “substance use” code and co-coded with the “challenges/gaps” code. Realizing the emergent themes’ alignment with the CCM, we organized them under the headings of the CCM’s core elements to report on our findings in the Results section.

(b) Codes: A codebook was iteratively developed by the study team. The first step in this process was creating a brief summary of each interview, highlighting the main topics discussed by the participant. These topics guided the initial development of the codebook. Three study team members independently coded three interviews, then discussed their codes until consensus was reached on the meaning and application of the codes. Additional codes emerging from the discussions were incorporated into the codebook. Each of these three researchers was designated as the primary coder for approximately a third of the remaining interviews. Any passages that were difficult for the primary coder to assign codes to were discussed as a team. New emergent codes were added to the codebook as needed throughout the entire coding process. We adhered to the widely-used concept of saturation (i.e., the point at which additional data do not give rise to new themes) (Strauss and Corbin 1998; Guest et al. 2006; Charmaz 2014), which we reached after approximately ten interviews each for the veteran participants and the stakeholder participants, to guide our decision to not collect additional interview data. We used the NVivo 11 Qualitative Data Analysis software (NVivo Qualitative Data Analysis Software 2012) to capture all coding activity.

(c) Themes: With the NVivo software we generated reports of data segments coded with the “mental health” code and/or the “substance use” code and co-coded with the “challenges/gaps” code. We thematically analyzed the reports (Miles and Huberman 1994) to identify emergent themes directly from the interview data on participants’ experiences and perceptions of coordination needs of veterans with MHDs and SUDs being released from incarceration. Throughout the analysis, we kept in mind that we had used purposive sampling for participant recruitment, which is meant to lead to illustrative inferences about what is possible (unlike probability sampling for quantitative studies, which leads to drawing statistical inferences about the prevalence of specified possibilities) (Wood and Christy 1999; Chang et al. 2009). We were thus careful not to characterize our findings based on the frequency with which each theme is mentioned by participants (i.e., the number of interview participants linked to a finding), beyond confirming the frequencies only to ensure that all data are accounted for (Sandelowski 2001; Chang et al. 2009). As the themes emerged, we realized their alignment with the CCM. Specifically, we noticed that the coordination challenges emphasized by the themes were similar in nature to clinical coordination challenges that the CCM has been found to effectively address (LaBelle et al. 2016). We therefore organized them under the headings of the CCM’s six core elements as reported in the results below.

## Results

The interviewed veterans were incarcerated for 11.5 years on average (SD = 12.5 years), and five of them were incarcerated for a sexual offense. Their mean length of military service was 3.2 years (SD = 2.8 years), and they represented the Army, Navy, and Marine Corps military branches. Four of them had combat exposure during their military service. Of the interviewed stakeholders, nine, six, and seven were from federal, state, and local organizations, respectively. Eleven of them served in supervisory rather than front-line reentry support roles, and ten of them were female.

Table 2 summarizes the emergent themes from our analysis, arranged by their CCM element that can be operationalized in the reentry context to help address the challenges identified by the themes. We describe below our findings in further detail for each CCM element, and also provide examples from our interview data that are related to each element. We use “veteran participant” to refer to reentry veterans we interviewed and “stakeholder participant” to refer to interviewed representatives from Massachusetts organizations involved in the reentry process.

**Table 2:** Summary of emergent themes from interview data by each core Collaborative Chronic Care Model (CCM) element

Core CCM element	Summary of emergent themes from interview data
Work role redesign (Structuring care tasks of multiple clinical staff in relation to one another, such that patient needs are collaboratively anticipated and met in a timely manner)	Coordination challenges among organizations involved in veterans’ reentry – e.g., addressing mental health and substance use disorder needs while meeting legal requirements
Patient self-management support (Strengthening patient’s ability to effectively contribute to his/her own wellbeing even during times when he/she is not in direct contact with care providers)	Veterans’ fear of reentering society – e.g., their ability to get housing, avoid substance use relapse, and address mental health symptoms
Provider decision support (Furnishing relevant information to care providers about available services, treatments, and expertise, to help them best address patient’s care needs)	Uneven knowledge by reentry support providers regarding available services when deciding which services to connect a reentry veteran to and whether he/she is ready/willing to receive services
Clinical information systems (Activating feedback systems to share data and monitor both how care is being delivered and how patient is responding)	Lapses in mental health or substance use disorder medications between release and a first scheduled health care appointment, as well as challenges in transfer of medical records
Linkages to community resources (Connecting patient to care resources beyond those available from his/her main clinical setting)	Inconsistent awareness of existing services and resources available across a disparate reentry system
Organizational / Leadership support (Championing of clinic’s change efforts toward more CCM-oriented care – i.e., care exhibiting core CCM elements – by clinic’s organizational leaders)	Reentry plans designed to address only immediate transitional needs upon release, which do not always prioritize mental health and substance use disorder needs

### Work role redesign

Reentry involves multiple organizations’ interactions with one another and with the veteran being released from incarceration. Participants often mentioned the difficulties faced in simultaneously meeting the release and reentry requirements of each organization, especially while addressing the mental health and/or SUD needs of the veteran.

For veterans, addressing MHDs and SUDs (commonly co-occurring in individuals) may require different state departments. The Department of Correction and the Department of Mental Health have different procedures for sharing information about veterans awaiting release. This makes timely coordination a major challenge, for instance holding a MHD or SUD housing space for a veteran whose release date may shift, on short notice, by several days or even weeks. Coordinated procedures to address veterans’ physical health care needs are reasonably established, but this is not the case for mental health care needs. A stakeholder participant noted in terms of coordinating housing in light of health care needs: “And I’ve had a good working relationship with [the health care team of the individual released from incarceration] for like the medical piece. But the mental health piece kind of falls apart.”

Coordination challenges persist after release. A veteran may have post-release mental and physical health care referrals in place, but programs have difficulty tracking whether the appointments were kept. Veterans, similarly, reported challenges encountered in coordinating treatment requirements, such as group therapy sessions, with their terms of release that may involve frequent appearances for probation, parole, or court. A veteran participant mentioned, “… I was just about to go into [an SUD treatment program] and the [district attorney] wanted me to … do [more time] … they pulled me out of the program to go to jail … [it] was crazy.”

### Patient self-management support

During reentry, an individual transitions from a regimented environment, with reduced choices and decision-making, to one in which there may appear to be few requirements and an overwhelming number of choices. This transition can be especially salient for veterans who may have spent many years in the controlled military environment prior to incarceration.

The fear of transitioning to a less controlled environment may be accentuated for veterans with MHDs and SUDs. One veteran indicated that he had had a support system, of other inmates while incarcerated, which prevented the return of his depressive symptoms. He noted the relative absence of such support post-release, and was concerned that he would have difficulty with managing his mental health care needs. Other veterans described being placed in transitional housing post-release, where there was active substance use among residents, indicating the important role of the environment in facilitating or impeding self-management of MHDs and SUDs.

Participants also mentioned the help veterans need in building skills to become independent of programs. VA domiciliaries (combined transitional housing and SUD recovery centers) accept veterans with MHD and SUD histories, but during their time there veterans should be developing ties to and support in the broader community. A stakeholder participant expressed, “So the hope is we’re trying to help them build a life out there so that they will only be coming back maybe for the psychiatric visit or when they need some sort of quick tune up.”

### Provider decision support

Reentry is complicated by the policies regarding which supportive services are available, or restricted, for which veterans leaving incarceration. Providers of health care and support services need to know such things as the type of offense (e.g., sexual offense), history and status of MHDs and SUDs, housing availabilities, and treatment options.

A stakeholder participant noted that organizations involved in reentry services need a better understanding of which services are available to which veterans. For example, treatment for persons with sexual offenses is limited in VA – thus providers must know where those services are, and the eligibility requirements. A stakeholder participant shared an example of information that ought to be more readily known by providers: “… [a non-VA treatment organization] – all they do – their population is sex offenders, strictly.” In contrast, VA has many services to treat MHDs and SUDs that cannot refuse to treat, due to recent incarceration, otherwise eligible veterans. Yet some stakeholder participants perceived that some VA programs take all veterans, whereas others will not accept veterans with recent incarceration history. Without the “insider knowledge,” participants’ perception was that reentry planning by providers of health care and support services is necessarily imperfect due to lack of comprehensive understanding of what programs serve which type of veterans, resulting in frequent trial and error.

### Clinical information systems

Reentry is accompanied by a change in the treatment regimens and health care providers that a veteran with MHD or SUD would have had established while incarcerated. This kind of abrupt change poses challenges to the delivery of treatment and services after release, especially when multiple organizations or providers may be involved.

The involvement of multiple correctional, community, and VA systems in veterans’ reentry makes continuity of services particularly difficult. As noted by a stakeholder participant, “… [reentry veterans] are often released with two weeks of meds and have nowhere to go with them ….” The need for continuation of medication and therapy is well understood. However, there is limited sharing of health records across the systems that are involved (especially between prison electronic health records and the records of private or government health care systems), which is confounded by separate privacy and sharing policies. Another stakeholder participant expressed, “… [an individual being released from incarceration] might have a whole slate of medications that they’re on … that information is information that [the stakeholder’s organization] can provide. We obviously still have to abide by the same privacy practices as in any other case so there are times when we have it to provide but can’t provide it.”

### Linkages to community resources

Limited resources being available to attend to veterans’ reentry needs becomes a greater challenge when there are differences among involved organizations’ perceptions and reality of which services and resources are available and appropriate for whom. Also, services are often highly specialized, where housing needs are attended to by a different agency than medical, mental health, and SUD needs. Moreover, some programs are heavily grant-funded, which may lead to abrupt stops and starts of services.

Even when community-based services are available, they may not be utilized if its availability is not known to VA and veterans. A stakeholder participant running an outside-VA program for posttraumatic stress disorder treatment questioned why the program is not more widely publicized, noting that, “50-60% of our veterans would say that [the] program has been positive. Over 4,000 vets have been taken into the program in the different states we have been in.”

Community-based services may also not be utilized if they do not meet veterans’ needs. Stakeholder participants mentioned that disparate perceptions of where veterans ought to be receiving services also hamper seamless coordination of community resources. There was a misperception, for example, that all veterans had complete access to all VA services [when in fact veteran eligibility depends on income, combat experience, service-connected disability, etc. (US Department of Veterans Affairs 2019a)]. With shortages of detoxification beds at VA, participants expressed even greater concern that misunderstandings of available resources pose a threat to coordination for reentry.

### Organizational/Leadership support

Veterans with MHDs and SUDs leaving incarceration need coordinated management and support over a long period following release. This is particularly important for preventing relapse and worsening of mental health symptoms.

Participants noted the need for such close attention and coordinated check-ins. A veteran participant shared a personal experience of needing these check-ins: “… [check-in calls asking,] ‘How you doing,’ and that’s important. Because what happened is I told, this is, my case manager would call me and when I was doing well, I would answer the phone and be talking to her and then I would come in and make my appointments. But I told her, like when I’m not answering the phone, there’s usually something going on. That’s the first key indicator. I can look back and see, ‘Okay, I’m starting to isolate, I don’t want to take phone calls.’”

Participants mentioned that these continued check-ins are especially needed if a veteran’s mental health needs may not have been appropriately addressed while incarcerated. Unfortunately, there are limited spaces for veterans to receive longer-term mental health and/or SUD treatment, as voiced by this stakeholder participant, “… [reentry veterans] transition out to the community sometimes to no services of ours other than just to case management or phone contact.” This was concerning to this participant in that it may lead to increased risk of recidivism. Participants felt that these longer-term care issues could be better addressed if there were stronger organizational and leadership support to dedicate organizational resources toward programs such as longer-term peer support and case management for reentry veterans with MHDs and SUDs.

## Discussion

Our analysis identified several challenges to continuous/coordinated care of reentry veterans with MHDs/SUDs. Participants noted considerable variation among transitional housing programs, in the extent that they addressed mental health and SUD needs. Veterans’ fear of reentering the society was mentioned by both veteran and other stakeholder participants, especially for veterans less experienced in managing health care and more general life skills (e.g., opening bank accounts) on their own. Participants mentioned the incomplete knowledge available to reentry support providers when deciding which services to connect veterans to. A major challenge was medication/treatment continuity, critical for veterans with MHDs/SUDs. This was hampered by incomplete information about veterans’ correctional health records, medication regimens, and the quantity of medication on hand at release. Participants voiced the difficulty of maximizing on available resources from multiple reentry support organizations, due to inconsistent awareness/existence of such services. Importantly, reentry plans often do not address longer-term needs of reentry veterans. Even when such plans exist, there are few supportive resources to ensure their execution (e.g., sufficient case management, available transportation, and veterans’ planning skills).

The CCM has recently guided the delivery of anticipatory, coordinated, and interdisciplinary care across multiple VA medical centers (Bauer et al. 2019). This prompted our consideration of its application to the delivery of mental health and SUD services to reentry veterans. The model was useful in guiding our thought process in moving from the emergent themes toward identifying ways to enhance the coordination/integration across state, county, and community organizations, which have varying roles in delivering services to reentry veterans with MHDs/SUDs. Aligning to the CCM’s six elements (noted in parentheses), our analyses identified the need for (i) coordinated roles/responsibilities across reentry services (work role redesign); (ii) support for veterans to manage anxiety associated with community reintegration (patient self-management support); (iii) better needs-based matching of veterans to providers with relevant expertise (provider decision support); (iv) information systems to track medication/treatment records between jail/prison and civilian healthcare systems, especially for MHDs/SUDs, to avoid gaps in medication taking (clinical information systems); (v) more shared knowledge with/among reentry veterans regarding community resources (linkages to community resources); and (vi) dedicated organizational resources toward longer-term peer support and case management for reentry veterans with MHDs/SUDs (organizational/leadership support).

Table 3 outlines potential means of addressing identified challenges, guided by the CCM. There is already an infrastructure in VA to support reentry veterans with MHDs/SUDs (far right column). Nevertheless, the challenges (middle column) are substantial and unlikely to be met by these VA programs alone. Many VA medical centers, for example, have HCRV specialists (part of whose role is to facilitate reentry), and regional networks of VA medical centers (Veterans Integrated Service Networks) are also expected to have one or more staff members providing/coordinating reentry services.

**Table 3:** Potential means of and currently existent mechanisms for addressing identified reentry challenges, guided by the Collaborative Chronic Care Model (CCM)

CCM element	Potential means of addressing identified reentry challenges	Currently existent mechanisms for addressing identified reentry challenges
Work role redesign	Designate a lead case manager for the reentry veteran (among the various organizations involved with the veteran’s reentry), specifying backup procedures for when this case manager is not available	Veterans Justice Outreach specialists assess and manage cases of justice-involved veterans in local courts and correctional facilities; Health Care for Reentry Veterans (HCRV) services include outreach to incarcerated veterans and assessments prior to release
Patient self-management support	Enable the veteran to participate in planning his/her reentry and post-release daily routines, prepare the veteran for heightened anxiety to be faced, and provide him/her with the tools to self-advocate and seek support from clinicians even outside of pre-scheduled appointments	Some correctional facilities have reentry specialists; HCRV services include short-term case management upon release, including referrals to health care and social services
Provider decision support	Make available to all reentry support providers a regularly updated database of latest requirements, which can also be coupled with an electronic or telephone-based portal through which clarifications and questions can be posed to obtain real-time support in deciding services and programs to plan for	Some correctional facilities, interim housing facilities, and reentry support programs individually have knowledge regarding available resources for veterans leaving incarceration, and some have built regular communication with local HCRV specialists to be updated on latest changes
Clinical information systems	Establish information systems that serve as a comprehensive registry of all reentry cases and can be programmed to generate alerts for cases that are particularly at high risk for lapses in treatment/medication, along with clear processes for regularly inputting and updating the information on each case	Some regional VA networks are utilizing databases that help HCRV specialists identify incarcerated veterans to provide outreach to
Linkages to community resources	Hold regular forums at which reentry veterans and support providers can share their latest knowledge of resources available in the community, augmented by clear documentation of the knowledge shared at these forums that is maintained in an easily searchable format (e.g., an online log)	VA’s homelessness programs, Veterans Service Organizations, community-based organizations that have contracts to assist veterans, and states’ departments of veterans’ services provide information on available community resources
Organizational / Leadership support	Develop processes for regularly and frequently engaging and updating organizational leaders on the current state of coordination, how changes to the current state can support the achievement of organizational goals, and what resources are needed from the leaders to accomplish the proposed changes	Support for HCRV program from national and regional VA leadership; individual VA medical centers decide on their specific level of effort dedicated to supporting veterans’ reentry, to balance available resources across multiple related (e.g., homelessness) services

Gaps are highlighted by viewing existing mechanisms alongside potential means of addressing identified challenges. For work role redesign, it is unclear what happens when initially designated case management is temporarily or no longer available (e.g., due to career transitions of case managers). For patient self-management support, short-term case management may not suffice for reentry veterans needing longer-term mental health and/or SUD help, given that individuals with MHDs/SUDs may be more vulnerable than others in unstable environments into which they are released (Henwood et al. 2018). For provider decision support, individual programs’ knowledge of available services, and their communication with local HCRV specialists, can benefit from being more systematically shared/updated across the programs. For clinical information systems, there is not yet an established mechanism through which health information on incarcerated veterans are made available to physical/mental health providers to be treating them after release. For linkages to community resources, an efficient way for veterans and reentry support providers to look across the spectrum of all available resources, especially to find resources that would optimally complement one another, is desirable. For organizational/leadership support, regular communication paths, both from front-line reentry support providers to leaders, and vice versa, are important to dynamically determine what tangible organizational support is needed by providers to feasibly implement the reentry support that is envisioned by the VA organization as a whole.

A recurring theme is that existing information/knowledge needs to be systematically shared/communicated across the multiple reentry support entities. Based on this work, our aforementioned larger PIE project (Simmons et al. 2017) seeks to have veteran peer support specialists, ideally with some past criminal justice system involvement of their own, serve as coordinators among the different entities (in addition to the extensive individual-level support they provide to reentry veterans with MHDs/SUDs) (Kim et al. 2019). We recognize that this is but one modest step towards a systems/organizational change approach to improving reentry services.

The recurring theme here of needing shared, accurate, and up-to-date knowledge about resources is very pertinent to non-veterans leaving incarceration. Especially with regards to justice-involved populations with MHDs and SUDs, a number of recent studies focus on how their individual needs can be better assessed and met through process changes across the multiple organizations that they interact with. For instance, Kopak and colleagues’ study (2019) highlights the need for jails to conduct behavioral health assessments with an SUD emphasis, to connect rural inmates to programming options that can help prevent reincarceration. For individuals with opioid use disorders in particular, Reichert and Gleicher’s study (2019) indicates the need for more up-to-date training of probation department staff to enable interagency collaboration that can help link the individuals to evidence-based treatment, which can then lead to their better probation requirement adherence and importantly positive outcomes such as reduced recidivism. Furthermore, support for individuals with MHDs in the justice context overall, from transitional housing (Heard et al. 2019) to needs during parole and probation (Bryson et al. 2019), are receiving increased attention in the field.

Our findings suggest that the CCM complements the Sequential Intercept Model (SIM) (Munetz and Griffin 2006), which provides an outline of the entire criminal justice system and indicates points for diverting individuals and keeping them out of the system. Our work focuses on the last two intercepts of the SIM – Reentry and Community Support, by turning to the CCM for potential coordination mechanisms that can address the specific needs of both veterans and non-veterans moving through these last two intercepts of the SIM.

The CCM has a robust evidence base for the effectiveness in care for MHDs/SUDs that are structured around its elements (Miller et al. 2013; Woltmann et al. 2012; Badamgarav et al. 2003; Gilbody et al. 2006; Alford et al. 2011). Strategies for successfully implementing the CCM across a variety of care settings are being increasingly developed/tested (LaBelle et al. 2016; Kilbourne et al. 2007; Wiltsey Stirman et al. 2012). Common barriers/enablers regarding CCM-based strategies and their implementation have been extensively explored by such works, which CCM-based approaches for enhancing reentry for MHD/SUD individuals can learn from and adapt. For instance, (i) administrative obstacles of tracking individuals’ health records, and (ii) limited resources for following up with those individuals, were barriers identified/addressed by LaBelle et al.’ 2016 study to implement the CCM for opioid treatment (LaBelle et al. 2016). That work’s CCM-based strategies included (i) supporting an increased information system capability for tracking and (ii) creatively educating/engaging staff not traditionally associated with treatment. These strategies can be adapted for use by quality improvement efforts targeting similar challenges identified by our study under the clinical information systems and work role redesign elements, respectively. Accordingly, future studies should assess, specifically for justice-involve populations, to what extent it is important to have particular combinations or all of the CCM elements addressed for effective reentry support.

We are fortunate to be able to compare our findings to two recently published systematic reviews that summarize research on reentry initiatives that are not specific to the veteran population. Berghuis’ review (2018; of reentry programs for adult males) found the need for (i) continuity of care, (ii) clear communication between involved institutions, (iii) cooperation from the community, and (iv) focus on long-term needs. These are very much in line with the emergent themes from our study as outlined in Table 2, respectively under the core CCM elements of clinical information systems, work role redesign, linkages to community resources, and organizational/leadership support. Kendall and colleagues’ review (2018; of qualitatively evaluated reentry programs addressing problematic drug use and mental health disorders) reported on the importance of ensuring (i) continuity of care, (ii) role of case workers, (iii) access to needed services, and (iv) personalized approaches to case management. Again, these are closely in line with the emergent themes from our study, respectively under the core CCM elements of clinical information systems, work role redesign, linkages to community resources, and provider decision support. Less highlighted in these recent reviews of non-veteran-specific reentry efforts was our emergent theme under the core CCM element of patient self-management support – i.e., the need to strengthen the veteran’s own ability to get housing, avoid substance use relapse, and address mental health symptoms. This difference may be related to how veterans, when considering their time in the military in addition to while incarcerated, are likely to have spent more years within a regimented environment that did not require them to often personally evaluate choices and make decisions. This may mean that veterans need additional support in self-management as they reenter into the community, where less requirements and more choices could be overwhelming.

This is especially true for navigating the landscape of various community services that support reentry, provided by multiple social, justice, and healthcare organizations that are often limited in their coordination with one another, as our findings indicate. Smith and colleagues, in their recent narrative review of community services for justice-involved women (2019), provide a comprehensive conceptualization of how each of these social, justice, and healthcare systems belong within the overarching community health delivery system (CHDS). They found CHDS to be fragmented and in need of better integrated service delivery, which suggests that many of our identified reentry challenges, and potential relevance of the CCM in addressing them, are likely applicable beyond the veteran population.

Our study is limited in its geographic reach, having been conducted within the context of our larger PIE project’s efforts to implement peer-based services in Massachusetts to support reentry veterans (Simmons et al. 2017). But given that we selected study participants to represent a broad spectrum of veteran, federal, state, and community perspectives, we expect the identified themes to remain highly applicable to the field. Our and other future studies, while being attentive to new themes, should also examine how the CCM aligns with existing models of reentry coordination [e.g., Critical Time Intervention for supporting reentry for individuals with mental illness (Draine and Herman 2007)], to ensure that the CCM enhances, rather than replaces, models that have shown some effectiveness. This directly echoes the compelling case made by Finlay and colleagues in their recent review of health and healthcare for justice-involved veterans (2019), that what is needed is not more models, but rather a consolidated common framework using which the various research efforts to improve health and well-being of justice-involved populations can be understood in relation to one another.

There are additional potential limitations to this work that should be noted. First, although we observed saturation in emergent themes to guide our decision to not collect additional interview data, there is always the possibility that more interviews could have revealed additional findings. However, we paid careful attention to abiding by established qualitative research methodological guidance (Strauss and Corbin 1998; Charmaz 2014) in carrying out the researcher’s responsibility in deciding the adequate sample size. We also recognize the drawbacks inherent to the grounded thematic analysis approach [i.e., (i) initial inductive consideration of themes being less structured than for deductive approaches and (ii) subjectivity regarding how best to move from inductive to deductive considerations (e.g., aligning to CCM elements in our case) in the analytical process], which we accept as a tradeoff for the approach’s ability to bring context-aware participant insights to light (Chapman et al. 2015). Second, findings from our study may be rendered less applicable by unforeseen policy and/or other contextual changes since the interviews. To this end, we have closely consulted our study partners (both leadership and front-line reentry providers at each of federal, state, and community organizations) to ensure that they do not have concerns regarding the potential outdatedness of our findings, and we encourage our readership to pay particular attention to future contextual changes in considering the implications of our findings. Third, the authors’ experience in studying CCMs may have led to recognizing the connections between emergent themes and the CCM specifically, rather than to other care models less familiar to the authors. In addition to noting this reflexivity here regarding the perspective from which we are coming at the data (Cohen and Crabtree 2006), and although our data did not give rise to themes that are counter-CCM (i.e., where one or more CCM elements are perceived as being not useful, or harmful, for reentry support), we would also like to emphasize the need for future studies to formally test the effectiveness of reentry support interventions that are built around the CCM. Fourth, adhering to the quality improvement designation of this study, we did not collect additional demographic data that was not directly related to assessing the main quality gap of interest (i.e., current reentry challenges faced by veterans). Examining our findings alongside a more robust collection of sample demographics could have revealed additional specific patterns in our findings that are associated with particular subsets of participants.

## Conclusions

Reentry planning grows in prominence as federal, state, and county jurisdictions modify laws and regulations to reduce prison and jail terms and shrink incarcerated populations (Sentencing Reform and Corrections Act 2015). Moving forward, the evidence-based CCM can bring rigorous coordination and implementation approaches to this expanding field of reentry planning, particularly for persons with mental health and SUD needs. The CCM’s application may potentially contribute to reductions in mental health crises and overdoses in the precarious first weeks of the reentry period (Baillargeon et al. 2009; Meyer et al. 2011; Massoglia and Schnittker 2009), through enabling more effective coordination of care and services across correctional, community, and healthcare systems.

## Data Availability

Raw interview data are not publicly available due to them containing information that could compromise participants’ privacy. Derived data supporting the findings of this study are available from the corresponding author upon request.

## Abbreviations

CCM: Collaborative Chronic Care Model
CHDS: community health delivery system
HCRV: Health Care for Reentry Veterans
IRB: Institutional Review Board
MHD: mental health disorder
PIE: Post-Incarceration Engagement
SIM: Sequential Intercept Model
SUD: substance use disorder
VA: Department of Veterans Affairs
